# Making Sense of Uncertainty in the Science Classroom: A Bayesian Approach

**DOI:** 10.1007/s11191-022-00341-3

**Published:** 2022-06-14

**Authors:** Joshua M. Rosenberg, Marcus Kubsch, Eric-Jan Wagenmakers, Mine Dogucu

**Affiliations:** 1 University of Tennessee, Knoxville, 1122 Volunteer Blvd, TN 37996 Knoxville, USA; 2 IPN–Leibniz Institute for Science and Mathematics Education, Olshausenstraße 62, D-24118 Kiel, Germany; 3 University of Amsterdam, Amsterdam, Netherlands; 4 University of California, Irvine, CA USA

## Abstract

Uncertainty is ubiquitous in science, but scientific knowledge is often represented to the public and in educational contexts as certain and immutable. This contrast can foster distrust when scientific knowledge develops in a way that people perceive as a reversals, as we have observed during the ongoing COVID-19 pandemic. Drawing on research in statistics, child development, and several studies in science education, we argue that a Bayesian approach can support science learners to make sense of uncertainty. We provide a brief primer on Bayes’ theorem and then describe three ways to make Bayesian reasoning practical in K-12 science education contexts. There are a) using principles informed by Bayes’ theorem that relate to the nature of knowing and knowledge, b) interacting with a web-based application (or widget—Confidence Updater) that makes the calculations needed to apply Bayes’ theorem more practical, and c) adopting strategies for supporting even young learners to engage in Bayesian reasoning. We conclude with directions for future research and sum up how viewing science and scientific knowledge from a Bayesian perspective can build trust in science.

**Supplementary Information:**

The online version contains supplementary material available at 10.1007/s11191-022-00341-3.

## Introduction

Uncertainty is ubiquitous in science. Consider, for instance, uncertainty because instruments cannot measure the thing they purport to measure with perfect precision or the uncertainty due to differences in observations made at different points in time (Fuller, 2009). Another form of uncertainty is its central role within explanatory theories—such as those associated with evolution and quantum physics—for which models take the form of probabilistic statements about the world (Gigerenzer, 2000). Negotiating these and other forms of uncertainty through constructively arguing—and building toward consensus while uncertainty is present—is a crucial part of the scientific process (Gigerenzer, 2000). It is also a key part of science education (Duschl, 2008; Manz & Suárez, 2018; National Research Council 2012; Nussbaum, 2011; Szu & Osborne, 2012; Thagard, 2000). Concomitantly, some philosophers of science have suggested that science is a process that builds increasingly better models which allow us to make increasingly more accurate predictions (Carnap, 1935; Feyerabend et al., 1975; Giere, 2010; Kuhn, 1962; Lakatos, 1976; Nersessian, 2002; Reichenbach, 1977).

Past research indicates that scientists, historians, and philosophers are aware of the uncertainty and limitations associated with reasoning from scientific evidence under conditions of uncertainty (e.g., Polanyi, 1962, 1966). Yet, scientific knowledge is often represented to the public as immutable (Carey & Smith, 1993; Duschl, 1990; Manz & Suárez, 2018). Trust in science can erode when scientists individually or collectively change their views, such as during the first phase of the COVID-19 crisis, when scientists faced severe criticism from the public, the media, and politicians for changing their recommendations based upon new scientific data and findings (Kreps & Kriner, 2020; van der Bles et al., 2020). A similar tension between how certain scientific knowledge is perceived and how uncertain it is concerns vaccines for COVID-19—a tension that involves reconciling robust but still initial clinical trial data and new data from population-level vaccination efforts. This tension also involves regulatory organizations, politicians, and medical, pharmaceutical, and scientific experts making policy-level recommendations (and individual decisions) amid uncertainty (Kreps & Kriner, 2020; van der Bles et al., 2020).

In this paper, we argue that we can address these challenges concerning trust in scientific knowledge by considering scientific knowledge not as correct or incorrect or true or false, but rather considering the degrees of belief that one might express considering prior information and new evidence. In other words, we argue for the use of a Bayesian perspective that emphasizes *subjective probability* (Batanero et al., 2016; Konold, 1991).

Notably, we argue that some of the challenges around trust in science can begin to be addressed by taking a Bayesian perspective on how people—and students—understand uncertainty. Thus, this paper is not about how scientists and statisticians might use Bayesian methods; existing books and articles address this topic (Gelman et al., 1995; Kruschke, 2015; Kubsch et al., 2021; Levy, 2016). Instead, we argue that science learners can use Bayesian tools to make sense of uncertainty more flexibly. Despite attention from statisticians and education researchers (e.g., Dogucu & Hu, 2021), there has been little research in the grades K-12 (or pre-collegiate) science education community on Bayesian methods. There are several notable exceptions to this dearth of research: two papers that adopted a Bayesian approach to a particular science practice, argumentation (Nussbaum, 2011; Szu & Osborne, 2012), and work in physics education that is at the undergraduate level. We build on and aim to extend this prior research by considering how a Bayesian approach can help learners make sense of uncertainty and reason scientifically in K-12 classroom contexts—above and beyond their involvement in argumentation. Indeed, our focus is more on analyzing and interpreting data, and that of developing and using models, both core science practices (Lehrer & Schauble, 2015; National Research Council,  2012) that we argue can be bolstered by considering a Bayesian lens.

We argue for the usefulness of Bayesian perspectives for science education by drawing on research from the work of individuals from diverse disciplines: (a) psychologists using Bayesian models of cognition (Gopnik, 2012; Tenenbaum et al., 2006), (b) statisticians and statistics educators advancing tools and practices for Bayesian methods (Albert, 2002; Bolstad, 2002; Hoegh, 2020; Hu, 2020; Martignon & Erickson, 2014; Sedlmeier, 2007), and (c) science education scholars who have advanced a Bayesian perspective on argumentation (Nussbaum, 2011; Sedlmeier, 2007) and scientific reasoning (Warren, 2018, 2020). In doing so, we claim that Bayesian reasoning can provide an intuitive way for people to think about data and evidence under uncertainty (Gopnik, 2012), including in classroom contexts (Leuders & Loibl, 2020; Nussbaum, 2011; Sedlmeier, 2007; Szu & Osborne, 2012; Warren, 2020; Witmer, 2017).

As this introduction has suggested, central to the contributions of Bayesian methods is viewing uncertainty *probabilistically*, as probabilities can provide a language for expressing the degree of uncertainty in our knowledge in a wide range of domains (Gopnik, 2012). Following this introduction, we expand on how uncertainty has been expressed in various domains through a review of prior research on uncertainty in science and science education in Sect. 2 and provide a primer on Bayes’ theorem in Sect. 3. Then, we provide an overview of strategies to make Bayesian reasoning practical for K-12 science teachers and learners in Sect. 4, discussing the potential contributions of a Bayesian approach in K-12 science classrooms in Sect. 5 and considering the implications (and limitations) of a Bayesian approach in Sect. 6.

## Research on Uncertainty and Bayes’ Theorem in Science Education

### Uncertainty in Science

Probability and uncertainty are ubiquitous in scientific methodologies, scientific concepts, and how science is communicated (Gougis et al., 2017). The practice of science often begins with measurement, which has a random component (Fuller, 2009) that arises from random variations in the measurement process. Measurement error limits the certainty that one can have in the measurements. Reducing that measurement error has been critical for many discoveries in science, such as the sorting out of the periodic table that has resulted in the representation as it is known today (Fontani et al., 2015). However, deciding on adequate statistical procedures to determine measurement uncertainty is not always easy, and it regularly sparks debate. For example, consider a recent study on the relationship between SARS-CoV-2 viral load and patient age by Jones et al. (2020) that was heavily debated in the scientific community (Frick, 2020) because the authors discretized a continuous variable, reducing statistical power.

Probability and uncertainty are not only integral to some science practices (such as analyzing and interpreting data; see National Research Council (2012)) but are also a part of explanatory models of and theories for scientific concepts. For example, in the context of Heisenberg’s uncertainty principle—more generally in quantum mechanics (Feynman, 1951)—confidence is limited not only by measurement error but in principle—even in the perfect experimental setup, there is no deterministic outcome. Instead, one must calculate the probability of each possible outcome using the Born rule.^1^ To this day, there is an ongoing argument about how to interpret the puzzling quantum mechanical phenomena such as quantum tunneling (Carroll, 2019).

Another example of how probability and uncertainty are central to scientific concepts and respective learning activities is evolution (Fiedler et al., 2019). Evolution is modeled as a probabilistic process as the emergence of new variations can only be described probabilistically. Furthermore, the continued survival of these variations with the population is governed by natural selection or random drift, which also escape a deterministic description and are thus modeled probabilistically. Like quantum physics, how to interpret this probability has also sparked debate among researchers (Millstein, 2016).

Given that probability and uncertainty spark and sustain debate among scientists, it is perhaps unsurprising that misinterpretations happen when scientists in the natural and engineering sciences communicate about their findings with their peers or the public (Cumming, 2014; Gigerenzer et al., 2004; McShane & Gal, 2017). For example, error bars in the form of confidence intervals are routinely interpreted as distributions that assign a higher probability to the center of the interval (Kruschke & Liddell, 2018). Another example concerns very low *p*-values, which are sometimes misinterpreted as effect sizes (Gelman & Carlin, 2017; Nuzzo, 2014), although *p-*values describe how *incompatible* a set of data is with a set of assumptions. Furthermore, *p*-values and null-hypothesis significance testing (NHST) have been criticized for fostering black or white thinking: either accept or reject the null hypothesis, leaving little room for uncertainty even when there is (Aczel et al., 2018; Cohen, 1994).

In short, misunderstandings of statements about probability and uncertainty are common across the range of modes in which scientists communicate about their work—and these can lead to misinterpretations that can diminish trust in science and the process through which scientific knowledge is constructed (Kreps & Kriner, 2020; van der Bles et al., 2020). We next discuss the role of uncertainty not in science but in science education contexts.

### Uncertainty in Science Education

Probability and chance events play an important role in the life sciences (Garfield, 2003; Garvin-Doxas & Klymkowsky, 2008), particularly in learning about evolutionary processes (Tibell & Harms, 2017), and scholars have explored how knowing about probability might impact knowledge about scientific ideas. Recently, Fiedler et al. (2019) investigated the relationship between statistical reasoning and acceptance and knowledge about evolution in a large sample of post-secondary (University-level) students in the USA. They found that students’ statistical reasoning capabilities strongly predict both acceptance of evolution and knowledge about evolution. Research on teachers’ conceptions of evolution has suggested that exposure to curricular materials that emphasized the role of randomness in evolution bolstered teachers’ *acceptance* of evolution, but not their understanding of it (Nadelson & Sinatra, 2010). The authors conjectured that difficulties learners face when understanding probability and uncertainty may require extensive instruction.

In the physical sciences, students’ struggles with the probabilistic nature of quantum physics (Bao & Redish, 2002) and nuclear decay (Santostasi et al., 2017) have been established by prior research. Still, the underlying reasons and mechanisms remain less researched than in evolution. Research into students’ misconceptions has started to address this gap (Aguilar et al., 2014; Marshman & Singh, 2015; Stefani & Tsaparlis, 2009), and the findings mirror the position of Batanero et al. (1994). Specifically, the concepts of probability are taught abstractly and in a way that does not build upon the ideas that students already hold, the experiences that students have, and the language that students use. Thus, these studies affirm students’ struggles with probabilistic scientific concepts but hint at an underlying issue of more foundational challenges with reasoning about probability beyond science education contexts.

Adding to this challenging situation is that when scientists try to communicate their uncertainty, prior research has demonstrated that many people struggle to interpret the statistics (such as standard errors/margins of error, confidence intervals, and *p*-values that establish statistical significance) that are used to make inferences from scientific data (Gigerenzer et al., 2004; Sedlmeier, 2007; Tversky & Kahneman, 1974). Similarly, difficulties in understanding the technical language of probability can contribute to a struggle on the part of students when learning about concepts such as evolution or nuclear decay that are modeled in terms of probabilistic statements (e.g., Fiedler et al., 2017).

Bayesian perspectives have long been brought to bear on reasoning probabilistically. For example, the work of Tversky and Kahneman (1974) points to a range of biases that can be elicited by the language in which statements about probability and uncertainty are framed. A prominent example is the neglect of base rate information in medical testing. The likelihood of having a medical condition based on a positive test result is often overestimated because information about the (base) rate of people affected by the condition is not considered (Kahneman, 2012). This base rate can be viewed as a form of prior knowledge about how probable it is that a person has a disease before considering the test result. The neglect of base rate information means that diagnostic tests that return positive results may not indicate that the individual truly has the disease the test is intended to detect. This neglect of base rate information also manifests itself in the prosecutor’s fallacy (Thompson & Schumann, 1987).

Responding to and building upon Kahneman and Tversky’s work that suggests that people may not reason in a *proper* (or correct) Bayesian manner, Gigerenzer and colleagues provided essential insights into the underlying mechanisms for the occurrence of these biases. Their research suggests that the biases are at least partly the result of information about probability and uncertainty being provided in a way incompatible with the heuristics that people develop from their everyday experiences (Gigerenzer & Hoffrage, 1995; Jenny et al., 2018). Based on this premise, Jenny et al. (2018) demonstrated that students judge probability significantly better when language is adopted, aligning with the heuristics that most learners develop from their everyday experiences. In sum, it seems feasible to change representations of probability and uncertainty to better align with ideas and heuristics that learners hold to support, rather than hinder, learning.

Given that people struggle to understand probability, is the Bayesian account of reasoning under uncertainty a prescriptive rather than a descriptive account? We explore this question in the next section drawing on work from scholars studying children’s causal reasoning under conditions of uncertainty.

### Bayes’ Theorem and Research on How Children Learn to Reason Scientifically

In the past two decades, developmental scientists have used Bayes’ theorem to understand how children make sense of and reason about the world (Bonawitz et al., 2019; Gopnik, 2012; Tenenbaum et al., 2006). The notion that children (and people) weigh evidence about the world in a Bayesian way also builds upon, refines, and in some cases refutes aspects of much earlier work that documented the surprisingly complex ways that children could reason about the world (Piaget & Inhelder, 1969). Unlike earlier work on the stages through which children develop (e.g., the work of Piaget and other developmental *stage* theorists), recent work using Bayesian models considers the differences in the reasoning ability of children and adults to be a matter of degree, rather than one of a kind. From contemporary views of development, children hold initial ideas and understandings that they change (or update) in ways concordant with how Bayes’ theorem represents the updating of an initial belief in light of data (Gopnik, 2012).

In addition to developmental research that uses Bayes’ theorem to understand how children make sense of data and information in light of their initial ideas and beliefs, there is other, similar psychological work that does not explicitly adopt a Bayesian perspective. Specifically, research that studies how learners’ prior understanding influences probability judgments of different patterns in the data center on the same tension around interpreting data in light of initial beliefs that a Bayesian perspective highlights (e.g., Klahr & Dunbar, 1988; Masnick et al., 2007; Schwartz et al., 2007). For example, the work of Klahr and Dunbar (1988) considers the scientific reasoning process in terms of the two “searches” people undertake, of their beliefs and hypotheses in their mind and data and evidence collected or analyzed as a part of some investigation. Scientific reasoning involves the coordination of these two “problem” spaces, but neither entirely outweighs the other, like the Bayesian process of updating initial beliefs in light of data. We know that people—even children—can and do update their ideas in light of data, though the extent to which they do depends on many factors, including how much people could know about the topic, to begin with (Masnick et al., 2017).

In addition, prior research on learners’ perceptions of the plausibility of scientific explanations highlights the importance of the degrees of belief that learners hold about a particular scientific explanation (Lombardi et al., 2013, 2016). This work suggests the merit of a Bayesian perspective as a potential frame or theoretical account for (in these critical instances) analyzing patterns in data and conceptual change; it also has other benefits for teaching and learning.

Because of the advances made by research using a Bayesian perspective to understand human *development* (Gopnik & Tenenbaum, 2007; Gopnik & Wellman, 2012), one might conjecture that these ideas have been applied to education. For instance, Gopnik (2012) wrote that the use of Bayesian methods to understand child development can serve as “a scientific foundation for a long tradition on 'inquiry-based science education,” and that adopting a Bayesian perspective “could lead us to much more specific and scientifically supported proposals for education” (p. 1627). However, as science education scholars have bemoaned (Lehrer & Schauble, 2015), it has largely not been the case that “science itself could help turn young children’s natural curiosity and brilliance into better science teaching and learning” (Gopnik, 2012, p. 1627). Indeed, scholars have lamented that it is often obsolete applications of Piaget’s ideas (e.g., Piaget & Inhelder, 1969) that represent the most significant contribution of developmental ideas to education (Lehrer & Schauble, 2007).

At the same time that opportunities to connect developmental science with science education exist, scholars have emphasized the importance of students’ prior knowledge and understandings for their ability to update their understanding of scientific ideas (e.g., Lehrer & Schauble, 2004; Masnick et al., 2007, 2017; Schwartz et al., 2007). This highlights a potentially powerful connection between Bayesian methods and the priorities of science educators. Developmental and psychological research into Bayesian models of cognition supports and bolsters these educational efforts—especially science education efforts—to design instruction based on eliciting and understanding students’ ideas (Gotwals & Birmingham, 2016; Haverly et al., 2020; Windschitl et al. 2012).

There is an opportunity to use Bayes’ theorem and Bayesian ideas to bring together and to formalize a collection of ideas that are relevant to science teaching and learning. However, a Bayesian perspective—with a few exceptions (e.g., Nussbaum, 2011; Szu & Osborne, 2012)—has been primarily absent from research in science education; we discuss this argumentation-focused research by science education researchers next. Moreover, a Bayesian perspective may also have a role in building trust in science by representing uncertainty in a principled yet flexible way—in this way, having a potential impact on people’s scientific literacy. Before elaborating on the education—and science literacy-related roles for a Bayesian perspective, we introduce Bayes’ theorem from an accessible mathematical and statistical perspective (with connections to the axioms that follow from this perspective).

### Bayesian Approaches to Scientific Reasoning in K-12 Educational Settings

As we noted in the Introduction, the work of Szu and Osborne (2012) and Nussbaum (2011) both applied Bayesian perspectives to the science practice of argumentation. Szu and Osborne write that Bayes’ is useful as both a standard mathematical tool and a conceptual one; indeed, they write that considering the degrees of certainty in beliefs that individual students hold—different from applications of Bayes’ theorem that follow more or less deterministically from “external, objectively probabilistic systems” (p. 61)—is “the key leap that characterizes the debate about the value of Bayesian inference as a model of scientific reasoning” (p. 61). In this way, Szu and Osborne argue that the most significant use of Bayes’ theorem in science classrooms is as a model of informal scientific reasoning, aligning with similar (informal) approaches to inference within the statistics education research community (Batanero et al., 2016; Makar & Rubin, 2018). They offer some research-related backing for the use of Bayes’ theorem as well as some practical, instructional recommendations, some of which we detail later in this section.

Nussbaum’s (2011) work on Bayesian approaches to argumentation describes both an application of Bayesian methods in K-12 classroom contexts and ideas about how Bayesian methods can serve as an analytic framework for students’ argumentation. Concerning the latter, Nussbaum describes how the social issue of raising taxes to provide resources to homeless individuals was a rich context for students to engage in forms of Bayesian reasoning. In this application, Nussbaum describes how the prior and likelihood could be obtained from empirical evidence and how the estimates that result from applying Bayes’ theorem led students to re-evaluate their initial arguments.

The work of Szu and Osborne (2012) and Nussbaum (2011) begin to apply Bayesian ideas to science education and other relevant classroom contexts, making Bayesian ideas more vivid in the process. However, neither speaks to how a Bayesian perspective could have a role in bolstering understanding or trust in science by learners and others. More generally, these two contributions are steps toward conceiving what a Bayesian perspective may offer science education, but there remains room to build further. For instance, despite their association with statistical methods that are growing in use in post-secondary contexts and industry (McGrayne, 2011), no science education scholarship has applied Bayesian methods to another science practice: analyzing and interpreting data. We argue that this presents an opportunity to advance a Bayesian perspective and Bayesian reasoning within science education.

### A Bayesian Approach to Scientific Reasoning in an Undergraduate Context

To this point, we discussed past research on uncertainty in science and science education, research that explicitly takes uses Bayes’ theorem to understand children’s scientific reasoning and what approaches to argumentation look like from a Bayesian viewpoint. This section considers a pedagogical approach that leverages Bayes’ theorem in an accessible manner. We note that this research may appear like the research we reviewed in Sect. 2.5—on research that uses Bayes’ theorem to understand children’s scientific reasoning. But, that research uses Bayes’ theorem as a theoretical framework through which researchers can conceptualize and model scientific reasoning. Instead, the research in this section hands the Bayesian approach over *to learners*, asking what benefits such an approach to science teaching and learning may hold.

Specifically, in two studies intended to make Bayesian reasoning more accessible to students in college-level introductory physics classes, Warren (2018, 2012) implemented Bayesian updating activities into an introductory university physics course. These are important for supporting Bayesian reasoning in K-12 classrooms as they held to the tenets of a Bayesian approach but were also deliberately modified so that even introductory students could both use and understand Bayes’ theorem in the context of understanding physics phenomenon. The activities Warren designed were added to in-class and homework tasks. They generally asked students to evaluate their answers or results using Bayesian reasoning. For example, students were asked to record their initial confidence in the hypothesis they would test, asked to estimate how the data they collected aligns with their initial hypothesis, and update their confidence accordingly using Bayes’ theorem. In a quasi-experimental setting, Warren (2020) found that these activities positively impact students’ epistemic beliefs, including beliefs regarding the nature of scientific knowledge and the presence and importance of uncertainties.

Warren (2018, 2020) frames the Bayesian reasoning as part of a hypothetico-deductive process (Popper, 1979, pp. 30, 360). In this process, a model is evaluated by deriving a testable hypothesis and testing it against yet unknown data. Depending on the ratio between the likelihood of observing the data if the hypothesis is true and the likelihood of observing the data if the hypothesis is false, confidence in the model decreases or increases relative to the initial confidence in the model. This is analogous to our example with the Eastern Hemlock trees. The only difference is that we are no longer concerned with using data to learn about an uncertain parameter value (the proportion of infected trees) but concerned with using data to learn about a hypothesis where we are uncertain whether it is true or false.

Using Warren’s (2018, 2020) qualitative approach requires the rewriting of Bayes’ theorem (see Sect. 3 for the more common form) in the following way, where *θ* from the equation introduced in Sect. 4 takes the form of a hypothesis *H*, as represented in Fig. 2:

$$p\left( data\right)= \frac{p\left(H\right) \times R}{p\left(H\right) \times R + 1- p\left(H\right)}$$

*p*(*H*|*data*), or our posterior, is the confidence we can have in the hypothesis *H* after updating our initial confidence, or our prior, *p*(*H*) based on consideration of the new evidence, expressed in the updating factor *R* where:

$$R=\frac{p\left(H\right)}{p\left(\neg H\right)}$$

Note that *R* is equivalent to the predictive updating factor in our earlier example. Thus, *R* > 1 represents confirmatory evidence, while *R* = 1 represents inconclusive evidence, and *R* < 1 represents disconfirmatory evidence. Guidelines for choosing an adequate *R* exist (Kass & Raftery, 1995), e.g., 20 < *R* < 150 can be interpreted as the evidence strongly favoring H. *p*(H), the initial confidence in the hypothesis, ranges from 0 to 1, with *p*(H) = 0.5 representing maximum uncertainty, i.e., having no ideas about the validity of the hypothesis.

## A Primer on Bayes’ Theorem

Bayes’ theorem is a mathematical procedure to understand new evidence in light of prior information. In this way, updating what is already known is *mathematical. Still, Bayes’* theorem also includes an *epistemological* component—a component related to what is known about the world. What counts as prior information should be construed very broadly—it encompasses subjective judgments of how likely an event is and knowledge of empirical data from other related events.

The usefulness of Bayes’ theorem lies in how it presents a flexible yet principled way to update what is known in light of the evidence. Bayes’ theorem is not a cure-all; it is, instead, a formalization of something scientists and people alike already do: interpret evidence in light of what is already known. Moreover, it is epistemologically *normative* to the extent that it is the derivation of the laws of conditional probability.

As an example of the application of Bayes’ theorem, consider the following. The Hemlock Wooly Adelgid is an invasive insect that feeds on Eastern Hemlock trees, a tall-growing pine tree found in the Eastern USA. Because infestations can kill Eastern Hemlocks rapidly, in many affected areas, people take steps to protect Eastern Hemlocks that are affected by the Hemlock Wooly Adelgid; some systemic treatments can be effective (National Park Service, 2021).

**Fig. 1:**
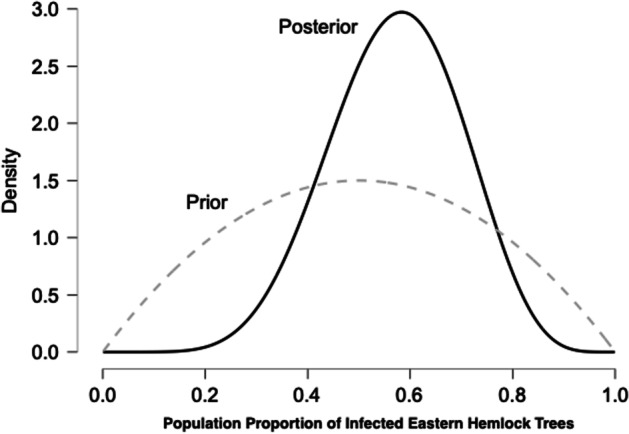
Example of using Bayes’ theorem to update prior information in light of new evidence. *Note.* A dome-shaped prior distribution captures the background knowledge concerning the proportion of affected trees. Observing ten trees (six affected and four not affected) drives a knowledge update that results in a bell-shaped posterior distribution. Figure based on the Learn Bayes' module in JASP.

A student (or a scientist) may be interested in the proportion of Eastern Hemlock trees within a specific area that exhibit signs of infection: diseased trees with visible white “cotton ball” clumps at the base of the needles of affected trees. Let us consider that in the location we are examining, each Eastern Hemlock tree grows about the same distance apart from every other tree (such that we may reasonably suppose the distance between trees to not be a critical factor to consider in an investigation). Before beginning an analysis, we need to establish what prior information we have: the relative plausibility of the different values for the proportion of affected Eastern Hemlock trees. Given what they learned in class and what they noticed walking into their school, the student’s best guess may be that about half of the Eastern Hemlock trees on their school playground are infected. They would not be surprised if the proportion of affected trees was between 30 and 70%, but—based on what they learned and observed—they would be somewhat surprised to find either no affected trees or that all the trees were affected. We can capture this background knowledge in the dome-shaped distribution shown as the dashed line in Fig. 1. This is the *prior* distribution, a crucial part of Bayes’ theorem. For now, consider just the prior. We will discuss what happens with the data next.

Now suppose a student starts to collect data by observing trees on their school’s playground. The first ten observations (with *Y* representing infected and *N* representing not infected) are {*Y*, *Y*, *N*, *Y*, *N*, *Y*, *Y*, *N*, *Y*, *N*}: six infected trees and four not infected trees. The question is how we can use this data to inform our knowledge about the proportion of trees that are probably infected. Here, Bayes’ theorem comes in. It states that at any point in time, our knowledge about the world after observing new evidence, the *posterior*, can be obtained by multiplying our prior information, the *prior*, with something *called a predictive updating factor* (Rouder & Morey, 2019; Wagenmakers et al., 2016):

Posterior = Prior × Predictive updating factor.

The predictive updating factor is the part where we plug in the data. To do so, it is helpful to express Bayes’ theorem mathematically in the following form (different from the form used by Warren [2018, 2020] that we introduced earlier):
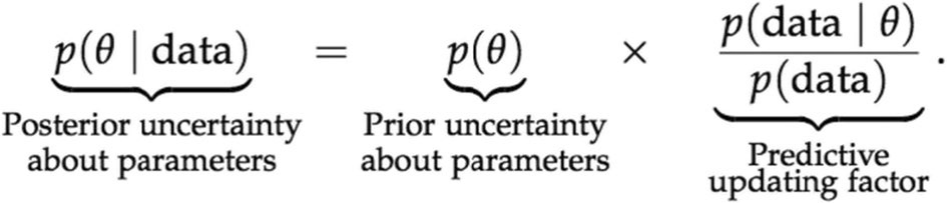


*θ* is some knowledge about the world that can take the form of a proposition, claim, hypothesis, or parameter value; ultimately, any account of the world about which we are uncertain (in our case, it is the proportion of probably infected trees). *p*(*θ*), the *prior*, represents our prior uncertainty about that knowledge (the dashed line in Fig. 1). The predictive updating factor consists of two components. First, *p*(data | *θ*) is the *likelihood*, that is, the extent to which the observed data were expected under *θ*, i.e., our prior knowledge of the world. Second, *p*(data) is the extent to which the observed data were expected across all possible values of *θ*. If *p*(data | *θ*) > *p*(data), the predictive updating factor will be larger than 1, meaning that our knowledge about the world *θ* predicts the data better compared to the average across all possible values of *θ*. Consequently, when we multiply the predictive updating factor with our prior, the value of our *posterior p*(*θ*|data) increases, representing reduced uncertainty of our knowledge about *θ*. Conversely, if *p*(data | *θ*) < *p*(data) our understanding of the world does not help to explain the data, the predictive updating factor will be smaller than 1, and the uncertainty of our knowledge about *θ* will increase.

What happens when we take our data and plug it into this equation? As the string of observations—six infected trees and four not infected trees—is more likely given our prior knowledge of *θ*, i.e., that the proportion of infected trees is somewhere between 30 and 70% and probably neither very low (below 10%) nor very high (above 90%), then when we average across all possible values of *θ*, i.e., those that include very low and very high probabilities, *p*(data | *θ*) > *p*(data) and the predictive updating factor is larger than 1. The value of our *posterior p*(*θ*|data) increases. Specifically, this results in the *posterior distribution* shown as the solid line in Fig. 1. The posterior distribution is more peaked than the prior distribution, indicating that the data have sharpened our knowledge about *θ*. In addition, the posterior distribution is higher than the prior distribution for values of *θ* between approximately 0.4 and 0.75 values of *θ* predicted the data relatively well and have therefore gained credibility. In contrast, values of *θ* lower than 0.4 and higher than 0.75 predicted the data relatively poorly, which is why they have lost credibility compared to the prior.

**Fig. 2:**
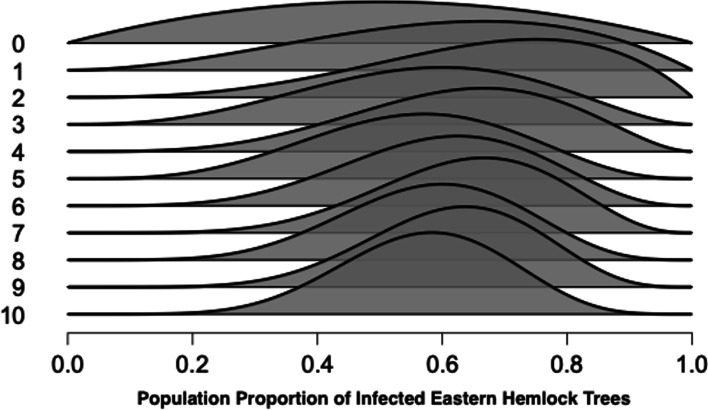
Example of sequentially updating what is known using Bayes’ theorem. *Note.* A dome-shaped prior distribution (line 0) captures the background knowledge concerning the proportion of infected trees. Each new observation results in an update to a posterior distribution, which becomes the prior distribution for the analysis of the next observation. Figure based on the Learn Bayes’ module in JASP.

Although we have demonstrated this updating process in a single step, it could also be executed sequentially, one tree at a time. For instance, the first observation we make is a *Y*, and in light of the prior, this makes the proportion of infected trees a bit higher. This slight change in knowledge is reflected in the difference between the distributions on the top two lines in Fig. 2; the line “0” represents the dome-shaped prior distribution, and the line “1” represents the posterior distribution after the first observations. Note that observing a *Y* has nudged the distribution a little to the right.

Crucially, the “nudged” posterior distribution after the first observation now takes the role of the prior distribution ready to be updated by our second observation. The second observation is again a *Y*, and line “2” shows that the resulting posterior distribution is nudged to the right once more. At every stage in the sequential updating process, the posterior distribution based on the observations seen so far becomes the prior distribution for incorporating the information from the following observation. Specifically, the changing distributions in Fig. 2 show our knowledge about the world is subject to constant change by making observations and integrating incoming information with current knowledge using Bayes’ theorem. It should be emphasized that although the updating process is conceptually different, the outcome is identical: the shape of the posterior distribution is unaffected by whether data arrive simultaneously or sequentially. We expand on this primer—specifically mathematical axioms that follow from Bayes’ theorem—in the Supplementary file.

## Strategies for Supporting Bayesian Reasoning in the Science Classroom

### *Strategy #1: Using Principles to Support Bayesian Reasoning*

If science instruction progresses *qualitatively*—our assumption for most K-12 science classrooms—the features of Bayesian reasoning can help bring a sense of coherence to the many ways in which uncertainty is manifest when students engage in scientific practices (e.g., National Research Council, 2012). Thus, we argue that Bayesian reasoning can provide this coherence as its distinctive feature by using the “concept of degree of belief to describe epistemic attitudes about uncertain propositions” (Sprenger & Hartmann, 2019, p. 2). Such a perspective reflects an embrace of the epistemic stance that scientific knowledge is not immutable but open to revision. In other words, scientific knowledge always comes attached with a degree of belief that can be updated following Bayes’ theorem’s rules when new evidence becomes available. The result of that updating process depends on the uncertainty of existing scientific knowledge, which is then expressed in the prior.

In this way, Bayes’ theorem applied to the scientific process, or *Bayesian reasoning*, emphasizes that science and scientific knowledge are always situated in context (Szu & Osborne, 2012) and society (Driver et al., 1994). These ideas about Bayesian reasoning can be captured in the following three principles that can be used as conceptual “tools” for teachers and learners:

1. *Be open to new evidence*. Scientific knowledge always comes with some uncertainty, and priors that denote absolute certainty (or impossibility) prevent scientific progress. This principle expresses the Bayesian degree of belief epistemology, exemplifying that scientific knowledge is tentative and that scientists should not make absolute or certain claims. Lindley popularized this idea in statistics and coined it “Cromwell’s rule” (Lindley, 1985, p. 104).
2. *Account for what is already known*. Evaluate new evidence in light of prior information. This emphasizes how scientific knowledge is not constructed in isolation but built upon earlier information and evidence.
3. *Consider alternative explanations.* Consider the evidence in terms of compatibility with all possible outcomes; in other words, consider counterfactuals. This expresses what in Bayesian philosophy is referred to as the Simple Principle of Conditionalization (Adams, 1965): When we weigh evidence, we must consider to what extent it supports the range of possible explanations for the data.

We think that emphasizing these principles can support students in connecting the frequent but often isolated references to uncertainty and limits of scientific knowledge in the *Framework for K-12 Science Education* (National Research Council, 2012) and the *Next Generation Science Standards* (NGSS Lead States, 2012). The descriptions of the science practices in the *Framework* (National Research Council, 2012) particularly contain expressions such as revising models, refining explanations, critiquing arguments, and considering the limitations of the precision of the data. However, what is lacking in these statements is an explicit and coherent rationale for why revising, refining, considering limitations is a necessary part—and a feature—of science, rather than a limitation or constraint of science. Familiarizing students with the principles as core components of Bayesian reasoning can foster ideas about scientific knowledge—epistemological ideas—from which the elements of the practices that pertain to uncertainty and the limits of scientific knowledge follow naturally. Moreover, this assumption is supported by the work of Warren (2018, 2020) which demonstrates that integrating Bayes’ reasoning into university science courses positively shifts students’ epistemic attitudes regarding the nature of knowing and learning. This assumption is also supported by research at the upper elementary (ages 10–11) grade levels (Kazak, 2015; Kazak & Leavy, 2018). We return to these three principles to support Bayesian reasoning that follow from Bayes’ theorem in the next section.

### Strategy #2: Using the Confidence Updater Widget

**Fig. 3:**
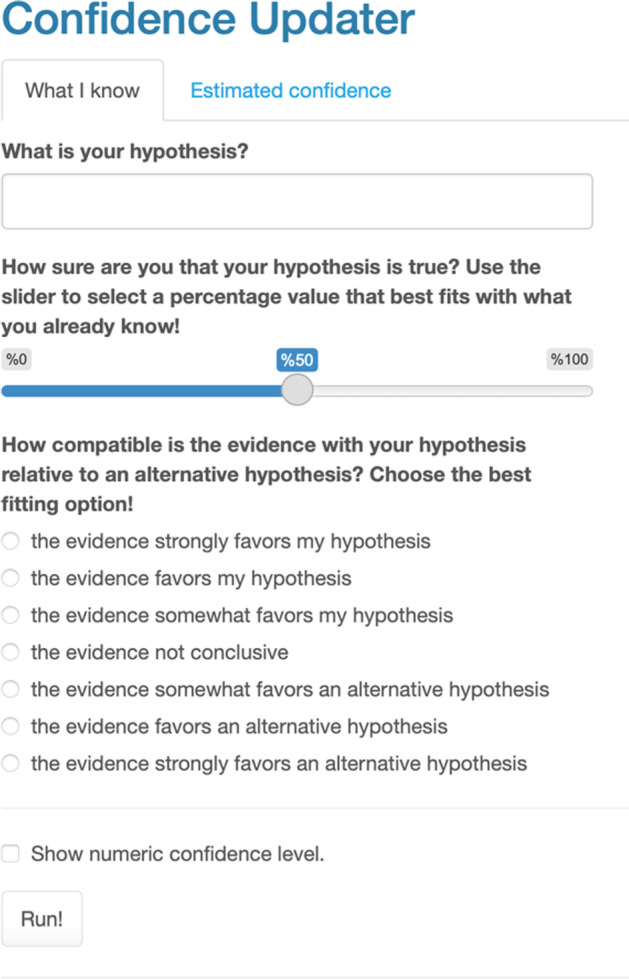
The Confidence Updater widget for updating one’s confidence in a hypothesis following Bayes’ theorem. *Note. p*(H) corresponds to the second question (How sure are you about your hypothesis?); *R* corresponds to the second question (How compatible is the evidence with your hypothesis relative to an alternative hypothesis?).

To support students’ focus on the conceptual elements of updating their confidence in a hypothesis, we build on Warren’s (2018, 2020) work by creating a *Confidence Updater* widget (a freely accessible, Internet-based tool). The widget is shown in Fig. 3, and it is available to anyone who wishes to use it at https://kubsch.shinyapps.io/Confidence_Updater/. This widget allows students to choose values for the strength of the new evidence—*R*—based on their interpretation of the evidence and *p*(H) based on their initial confidence. After performing the needed calculations, the widget returns a textual statement about updated confidence, *p*(H|E), in the hypothesis after considering the evidence and an optional numeric value for the confidence level.

**Fig. 4:**
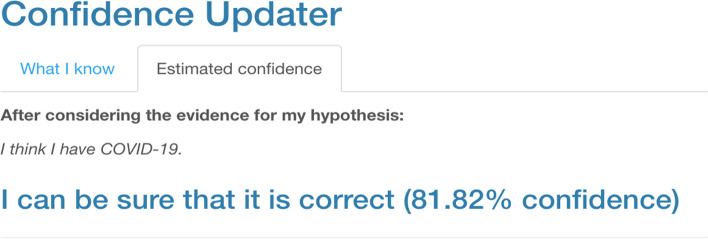
Example output from the Confidence Updater widget. *Note*. This output corresponds to the posterior distribution—the output from applying Bayes’ theorem.

Using the widget, one can obtain an estimate—based on Bayes’ theorem—for the confidence one can have in a hypothesis based upon the degree of one’s belief in the initial hypothesis—one’s prior—*p*(H), as well as the predictive updating power of the new evidence, *R*. One can see the example output in Fig. 4.

The three principles that follow from Bayes’ theorem (introduced in Sect. 4.1) are reflected implicitly and explicitly in the widget. The first principle that follows from Bayes’ theorem that we described, *be open to new evidence*, is emphasized as a natural consequence of Bayes’ theorem and the options absolutely certain that it is correct and absolutely certain that it is incorrect in the “How sure are you about your hypothesis?” section of the app. When students select from among these options, *p*(H) is set to 1 (certain that it is correct) or 0 (certain that it is incorrect), respectively. In such cases, neither the tentative (or changeable) nature of scientific knowledge nor the empirical basis of science is manifest. However, both are key elements of science (Abd-El-Khalick et al., 1998).

Consequently, whatever updating factor *R* students choose, the updated confidence will always be the same as the initial hypothesis—the prior—*p*(H) and considering the new evidence becomes pointless. In other words, no amount of data may change one’s belief in such cases. From this perspective, a question could be not scientific, as empirical evidence has no bearing on the answer to the question. If students selected any of the options that are equivalent to *p*(H) = 1 and then they observe strong contrary evidence and wonder how this does not affect the updated confidence are observing the evidence, a teacher could refer to the first principle—be open to new evidence—and point out to students how this degree of belief can inhibit any changes in beliefs even when confronted with strong evidence.

As Warren (2018) points out, “the fact that we can never reach a probability of zero is the quantitative expression of the maxim that we should never rule out any hypothesis with an absolute certainty” (p. 371). At the same time, even when the evidence is highly supportive of a hypothesis, the posterior probability will never equal precisely 1. In other words, there are no certainties where it comes to Bayesian epistemology. This reflects philosophical and historical accounts of science that emphasize its mutability over time (Kuhn, 1962), as expressed in principle 1.

The second principle, *account for what is already known*, appears in the wording of the prompt for selecting *p*(H) and becomes visible to students as it effectively moderates the power of the evidence. When prior beliefs and evidence align, the updated confidence is larger in comparison to either having no idea before or having prior beliefs that do not align. Students could notice this if they compare the updated confidence *p*(H|E) for students that had the same prior belief but evaluated the evidence differently or had different prior beliefs but evaluated the evidence in the same way. Again, as Warren (2018) says, “the fact that everyone starts with subjective prior probabilities and yet inevitably converges to a single asymptotic result illustrates the objective aspect of science” (p. 371), which can help build trust in science.

Finally, the last principle, *consider alternative explanations*, is reflected in how the prompt for *R* is worded. Furthermore, when students use the widget, they should be encouraged to argue for their choice of *R* and explain why they choose a specific option. In such an argument, students following the prompt should consider how their evidence supports their hypothesis and its compatibility with alternative hypotheses. That students must select the value for *R*, the predictive updating factor, is notable. Students must figure out the extent to which any single laboratory activity contributes to what we collectively know about a scientific phenomenon: As Warren (2018) writes, it leads to a more realistic view of what laboratory activities can accomplish (i.e., few single introductory physics experiments are likely to overturn established knowledge about the physical world, but they can lead to us updating our understanding)—as well as one that emphasizes scientific sense-making to a greater extent.

**Fig. 5:**
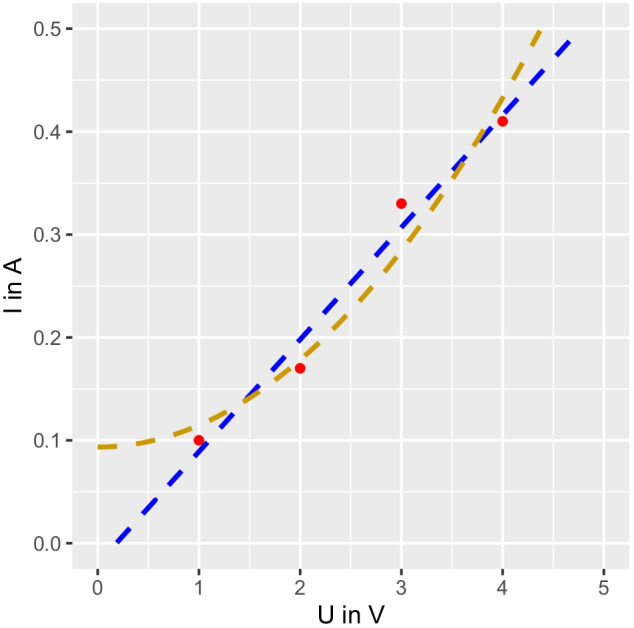
An example graph to illustrate how the Confidence Updater widget can be used. *Note*. The red lines represent data points from observations students could make. The blue line represents a student's possible explanation for how the current (*I*) relates to voltage *V* proportionally (or linearly). The yellow line represents a student's possible explanation for a higher-order (quadratic) relationship.

How does using the confidence updater change a typical science class activity? Here, we sketch how using the confidence updater can provide rich opportunities to discuss and reflect on epistemic attitudes. A standard activity at the middle grades’ levels is to understand the relations between amperage, *I*, resistance, *R*_E_,^2^ and voltage *V*, i.e., *Ohm’s law*, *V* = *R*_E_ ∗ *I*. To explore the relations reflected in Ohm’s law, students can measure the amperage in a circuit at different voltages, keeping the resistance fixed or constant. At the beginning of the activity, students can be asked to generate other hypotheses for the relationship between amperage and voltage. In our experience, students often propose a proportional (or linear), inverse, or quadratic relationship for this relationship—when resistance is held constant. Based upon their initial ideas—that the relationship is proportional, inverse, or quadratic, they may test their hypotheses using the widget. Specifically, following the second principle, students can now specify their prior confidence in their group’s hypothesis. Again, students may choose different priors based on their individual experiences. They may record several current measurements for different voltages and then graph the data. Figure 5 shows an example graph.

When students are asked to evaluate the evidence and set a value for *R*, they are primed to consider to what extent the evidence is consistent with their hypothesis relative to an alternative hypothesis, reflecting the third principle. Looking at the graph, one could see evidence for a proportional relationship as well as a higher-order positive relationship (blue and red dashed line in Fig. 5). Thus, the evidence is barely compatible with an inverse relationship between *I* and *U* but its supports both hypotheses that suggest a positive relationship between the variables. Thus, depending on what hypothesis students choose to investigate, they should select “data favors an alternative hypothesis” for *R* if their hypothesis is an inverse relationship and select “data somewhat favors my hypothesis” if their hypothesis is proportional or quadratic relationship. The updated confidence for every student will depend on their initial confidence—the prior (p(H))—in their hypothesis and the evaluation of the data, which will be different across groups. If students compare their results within groups, they should see how their initial confidence influences the effect of the evidence. However, if they repeat the activity, they should see how consensus is approached over a few iterations unless anyone chooses *p*(H) = 0 or *p*(H) = 1, which points, again, to the importance of the first principle (introduced in Sect. 4.1).

### Strategy #3: Supporting Bayesian Reasoning Among (Even) Younger Learners

We think that as accessible as the Confidence Updater widget may be for some students, *t* is worth briefly considering whether—and if so, how—younger children may engage in a Bayesian activity like that described above. Kazak (2015) and Kazak and Leavy (2018) demonstrate how Bayesian activities can be designed for younger students. For a study with fifth grade (10–11-year-old) students, Kazak (2015) created a game in which students drew one coin/token from each of two bags. If the colors of the two tokens matched, then students won. The question students were tasked to answer was, “Is the game fair or not?”. They played four different rounds of the game (using four different sets of bags).

Before playing the game, through a worksheet, students (a) articulated whether the game was fair and (b) their confidence in their beliefs about the game’s fairness. Three of the four games were designed to make determining their fairness challenging. For playing the games—and using a statistical software tool for learners, TinkerPlots (Konold & Miller, 2005)—students updated their beliefs about the game’s fairness. Their confidence shifted in conjunction with their analysis of data: both generally improved as students collected more data and simulated the collection of data through TinkerPlots.

Students first expressed their initial beliefs about the game’s fairness before playing it, updating these beliefs over time. As Kazak (2015) explained, “since these beliefs can change based on new evidence, it is important to assess the personal degree of confidence in the initial hypothesis or prediction and look at how it changes throughout gathering new relevant information” (p. 704). This work shows that students can express their *subjective probability* beliefs (Batanero et al., 2016; Konold, 1991) and—critically—update them over time. An instructional implication of this research is that educators can elicit students’ ideas about what they think about a phenomenon before conducting an investigation (and collecting data). This is an instructional practice already familiar to science educators and science teacher educators (Windschitl et al., 2018; Windschitl et al. 2012).

In addition to eliciting students’ ideas, educators can also prompt students to consider how confident they are in their beliefs or ideas. Reflecting upon and “updating” these beliefs can be supported throughout investigations (and data analyses) by prompting students to consider both their beliefs and confidence throughout an activity, which can help students to begin to understand the three principles that characterize Bayesian reasoning.

## Discussion

We have covered a lot of ground to this point, starting with prior research on uncertainty and Bayesian perspectives on science teaching and learning through a primer on Bayes’ theorem and three practical ways to introduce Bayesian reasoning to K-12 students. As we argued in the Introduction, the position for which we are advocating builds on and is intended to extend past research that makes a case for a Bayesian approach to argumentation (Nussbaum, 2011; Szu & Osborne, 2012) and prior work that brought a primarily qualitative, Bayesian approach to students’ analysis of data at the K-12 level (Warren, 2018, 2020). We extended this past work by considering Bayesian ideas’ impact beyond a single practice—argumentation—and adapting the work of Warren to be more accessible to and usable by K-12 science teachers and learners.

We acknowledge that the ideas discussed around the use of a Bayesian perspective in science classrooms may appear to be not directly relevant to science classrooms—at least classrooms at the grades K-12 grade levels. Moreover, Bayesian ideas may be too complex to teach students in these grades. We acknowledge these potential criticisms and point to how ideas that could be considered Bayesian are commonly deployed in most if not all science education contexts. For instance, many science educators consider it essential to understand students’ initial ideas before planning instruction (Haverly et al., 2020; Windschitl et al., 2012, 2018). There are also other examples of Bayesian ideas “hiding in plain sight” in science education. Conceptual change research makes similar points about the importance of recognizing what learners or individuals already believe before taking steps to persuade them to change their views or to explain new ideas to them (Lombardi et al., 2013, 2016; Nadelson et al., 2010; Sinatra et Al., 2014). Bayesian methods provide educators with a formal mechanism for working the initial ideas with which learners approach investigations and analyses of data into the claims learners can make—as a few studies have begun to demonstrate (Kazak, 2015; Kazak & Leavy, 2018; Warren et al., 2018, 2020).

Moreover, cognitive science research has demonstrated that learners’ initial ideas or familiarity with a topic or phenomenon impact their reasoning about it (Klahr & Dunbar, 1988; Masnick et al., 2007, 2017; Schwartz et al., 2007). Lastly, science educators have long sought to emphasize the tentative nature of scientific knowledge—even scientific theories (Abd-El-Khalick et al., 1998). But many of the approaches taken to analyzing data in science result in answers that can be seen by learners as correct or incorrect. A Bayesian perspective emphasizes this tentative nature of science and provides learners with a coherent approach for considering data and evidence when reasoning.

Considering how scientific knowledge can be presented to learners as immutable (Carey & Smith, 1993; Duschl, 1990; Manz & Suárez, 2018), it may be unsurprising that learners—and people—are dismayed when scientific knowledge that was once considered to be the consensus changes. When learners do not have a way to think about uncertainty, they may adopt an approach that considers scientific knowledge to be correct or incorrect—or for scientific sources to provide true or false evidence. From a Bayesian perspective, uncertainty about scientific knowledge can be viewed in terms of the degrees of belief that a person holds toward a belief, claim, or research—any prior information—in light of new evidence. We conjecture in this paper that such a paper can bolster individuals’ trust in science by providing a principled, flexible way to reason about uncertainty.

Indeed, a key potential benefit of Bayes’ may be that it provides a framework for helping students transition from their conceptual understanding of scientific ideas to understanding data (even when the pattern or signal in the data leaves us uncertain). In this way, we argue for the use of a Bayesian perspective that could influence research about (and teaching to support) learners’ epistemic considerations and how learners’ ideas change over time—factors that may have a bearing on how they understand science outside of school, or later as adults (Sinatra et al., 2014).

## Directions for Future Research

There are several avenues through which this can be approached in future research. We describe three specific directions for future research next. First, research on the role of Bayes’ in education has been chiefly carried out in the context of undergraduate statistics education, in which there have been reviews (Dogucu & Hu, 2021), recommendations, and calls to action (Dogucu & Hu, 2021; Gould et al., 2018; Hoegh, 2020), examples, design options (Albert, 2002; Bolstad, 2002; Gelman, 2008; Hu, 2020; Witmer, 2017) and debate (Johnson et al., 2020) over how to advance the place of Bayesian methods in undergraduate statistics and data science degree programs. Why has there been this attention? The accessibility of Bayesian methods in undergraduate classes is the result of advances in the necessary (for many uses) computer power (Gould et al., 2018) as well as the availability of tools that facilitate Bayesian analysis, especially for newcomers (Albert & Hu, 2020). As evidenced by the recent special issue of the *Journal of Statistics Education,* the pedagogy of Bayesian statistics is an active area of research in statistics education at the undergraduate level.

Second, we think learning progressions that center the practice of analyzing and interpreting data (and others, like developing and using models) informed by a Bayesian perspective could be helpful to create. As we have noted, analyzing and interpreting data is a core scientific practice. In line with this goal, the National Research Council’s (2012) *Framework for K-12 Science Education* emphasizes that “students evaluate the strength of a conclusion that can be inferred from any data set” and “recognize when data conflict with expectations and consider what revisions in the initial model are needed” (NRC, 2012, pp. 61–63). However, when people—across educational backgrounds—refuse to take the COVID vaccines because they judge them as unsafe (Sinatra & Hofer, 2021), it becomes painstakingly clear that science education often does not enable learners to analyze and interpret data in practical and meaningful ways. In line with researchers emphasizing the epistemic aspects of science teaching and learning (Krist et al., 2019; Berland et al., 2016; Duschl, 2008), we argue that a core reason for this lies in how contemporary science education engages students in analyzing and interpreting data does not support students enough in developing usable forms of the epistemology underlying this practice. In short, there is a disconnect in the *how* and *why* of engaging in analyzing and interpreting data, one that scholars have pointed to in the context of learners’ engagement in other science practices (Berland et al. 2016). We propose that building a learning progression for analyzing and interpreting data from a *Bayesian perspective* can provide a systematic connection between the *how* and *why* of analyzing and interpreting data.

Third, we think there are opportunities to research how a Bayesian perspective can support understanding and deploying what Berland et al. (2016) describe as epistemologies in practice. By doing so, students can learn*.* Specifically, a Bayesian perspective can support learners to understand key concepts about the nature of science—such as the tentative (or uncertain) nature of scientific knowledge (Abd-El-Khalick et al., 1998)—through their engagement in science practices. In this way, students may understand the nature of science and scientific knowledge *through engaging in science practices* that are more situated than if students learned *about* the nature of science (Berland et al., 2016). We also note that considering a Bayesian approach to students’ development of epistemological ideas invites (we think) constructive conversations about what is meant by the notion of cross-cutting concepts (National Research Council, 2012): A Bayesian approach could be associated not only with a *practice* (analyzing data, developing models, and others) but also as a type of cross-cutting concept (National Research Council, 2012) that can, in the context of learning about particular scientific ideas, unite the practice of argumentation with analyzing and interpreting data with the notions certainty and uncertainty. In this way, we think that Bayesian perspectives could inform ongoing conversations (e.g., Fick, 2018) bout the nature and contents of the cross-cutting concepts that inform science education standards.

## Conclusion

Even when science has made and continues to make significant advances, the history of science suggests that uncertainty will not be removed given scientific progress (Fara, 2010). Looking ahead, effectively navigating, counsel, and acting amid uncertainty may be as important as it has been at other uncertain points in history. Like the surprising utility of a Bayesian perspective in domains like education (e.g., developmental science), might an unexpected feature of a Bayesian perspective be that embracing uncertainty—equipped with the tools that Bayesian methods provide—makes individuals and societies more confident? In short, can we build trust in science by embracing uncertainty? We think these questions are *likely* to yield positive results for science educators and those advocating for a better-informed public.

## Supplementary Information

Below is the link to the electronic supplementary material.

Supplementary file1 (PDF 524 KB)

## Footnotes

1 See https://www.quantamagazine.org/the-born-rule-has-been-derived-from-simple-physical-principles-20190213/ for a primer on this.

2 We use *R*_E_ for electric resistance—instead of the traditional *R* to avoid confusion with the updating factor *R*.
