# Supplementary material for: Making Sense of Uncertainty in the Science Classroom: A Bayesian Approach
Source: Sci Educ (Dordr). 2022 Jun 14;31(5):1239–62. doi: 10.1007/s11191-022-00341-3 (PMC9196155; doi:10.1007/s11191-022-00341-3)
Supplement: Supplementary file 1 — Supplementary file1 (PDF 524 KB) [file 11191_2022_341_MOESM1_ESM.pdf]

Appendix A: The Epistemological Axioms that Follow from Bayes' Theorem

Integrating Inductive and Deductive Reasoning With the Bayesian Learning Cycle

Several years ago, physicists reported that they had observed elementary particles moving faster than the speed of light (Brumfiel, 2011)–thus defying Einstein's theory of relativity. However, instead of celebrating a historic discovery, the scientists started to look for what had gone wrong in their observations. With nearly a century of accumulated evidence that the theory of relativity holds, from a Bayesian perspective, scientists had a very strong prior belief about the speed of light. To change this belief, extraordinary evidence would have been required. Thus, a single instance of apparently faster-than-light elementary particles does not give a likelihood strong enough to change the prior in a meaningful way, and in consequence, the posterior–the knowledge about the world after making an observation–does not change in a meaningful way.

Rather than changing their belief about the speed of light, scientists looked for things that could have gone wrong with the measurement. This example shows how a qualitative take on Bayes" theorem can explain and predict many aspects of how animals (Okasha, 2013), people (Tenenbaum et al., 2006), and organizations (Kruschke et al., 2012) learn from observations. Recently, advances in computational power and techniques have started the more and more widespread application of this principle in a quantitative way that allows researchers to precisely express their knowledge about the world and then learn from observations.

Expressed in the Bayesian learning cycle (Figure 6), a Bayesian reasoning framework allows scientists to integrate the strengths of both, inductive and deductive research approaches, in a formalized way. Further, when specifying the prior, scientists openly and explicitly need to describe the uncertainty in the current state of knowledge, and the posterior captures how the data has changed the knowledge and the uncertainty in that knowledge. Thus, Bayesian reasoning is–in principle–open and transparent by design.

Mathematical Axioms That Follow From Bayes' Theorem

In this section, we build on the idea of the Bayesian Learning Cycle–that a Bayesian perspective aligns with a way of systematically taking in new information to improve what one knows (and how certain one is about what one knows–to elaborate on some general principles related to the nature of knowledge and knowing, or epistemological principles. We illustrate these to later show how they can have a role in the science classroom and how they can be used by individuals outside of classrooms and schools to understand and act upon scientific information.

In the previous section, we emphasized the idea that hypotheses, or ideas, gain credibility when they predict the data well, and lose credibility when they predict new data poorly (Wagenmakers et al., 2016). This idea generalizes to the "Fundamental Inductive Pattern":

> "This inductive pattern says nothing surprising. On the contrary, it expresses a belief which no reasonable person seems to doubt: *The verification of a consequence renders a conjecture more credible.* With a little attention, we can observe countless reasonings in everyday life, in the law courts, in science, etc., which appear to confirm to our pattern." (Polya, 1954, pp. 4-5)

The rule from Equation 1 includes the constant term $p(\text{data})$, which does not involve θ. As a result, Bayes' rule can be re-written as:

$$p(\theta \mid \text{data}) \propto p(\text{data} \mid \theta) \times p(\theta), \tag{4}$$

where '$\propto$' stands for 'is proportional to'. Thus, Bayes' rule states that our posterior knowledge $p(\theta \mid \text{data})$ is proportional to the likelihood $p(\text{data} \mid \theta)$ (or the extent to which the observed data are expected given θ) multiplied by our prior knowledge $p(\theta)$.

Equation 4 emphasizes that the posterior uncertainty about θ is a *compromise* between our prior uncertainty about θ and the predictive performance of θ. But, the posterior uncertainty after having observed the first datum becomes the prior uncertainty

for the next datum–and any other data (cf. Figure 2). Consequently, after having observed another datum, the posterior uncertainty represents a compromise of a compromise. As the data accumulate, the posterior uncertainty is more and more determined by predictive performance, and the impact of the initial uncertainty about θ is increasingly watered down: 'the data overwhelm the prior' (Wrinch & Jeffreys, 1919).

This implies (but does not dictate, as we discuss next) that a constant stream of accumulating data ought to bring any two people into an arbitrarily close agreement, no matter how divergent their opinions may have been at the outset. Two caveats exist. First and foremost, the data only overwhelm the prior if that prior is neither equal to zero (denoting an impossibility) nor one (denoting absolute certainty). If someone already knows for certain that a hypothesis is true or false, then you cannot adjust your opinion in light of the data: your initial opinion will be your final opinion, no matter what the data may indicate. Philosophically, adopting such priors is a dangerous practice; for instance, in antiquity, the adage of the *New Academy* –a school of skeptics headed by Carneades– was "never assert absolutely". In modern times, Lindley popularized this idea in statistics and coined it "Cromwell's rule" (Lindley, 1985, p. 104).

The second caveat is that, in real life, the data are sometimes relatively slow to overwhelm the prior, as people can be reluctant to change their beliefs (for a Bayesian model see Gershman, 2019). A more extreme case is *belief polarization*: confronted with the same stream of information, two people who hold different opinions may drift further apart instead of moving closer together (but see Anglin, 2019). This appears irrational, but several Bayesian accounts have been offered to explain the phenomenon (e.g., Cook and Lewandowsky, 2016; Jern et al., 2014).

Next, consider two specific values for θ, θ_1 and θ_2. We can use Bayes' rule to obtain

the posterior probability for each one:

$$p(\theta_1 \mid \text{data}) = \frac{p(\text{data} \mid \theta_1) \times p(\theta_1)}{p(\text{data})} \tag{5}$$

$$p(\theta_2 \mid \text{data}) = \frac{p(\text{data} \mid \theta_2) \times p(\theta_2)}{p(\text{data})}. \tag{6}$$

When we divide the posterior probabilities, the common term, $p(\text{data})$, can be dropped from each side of the equation, and we have:

$$\frac{p(\theta_1 \mid \text{data})}{p(\theta_2 \mid \text{data})} = \frac{p(\theta_1)}{p(\theta_2)} \times \frac{p(\text{data} \mid \theta_1)}{p(\text{data} \mid \theta_2)}. \tag{7}$$

We replace θ_2 with $\mathcal{H}_1$ (i.e., the alternative hypothesis, in which a test-relevant parameter ξ is free to vary) and θ_1 with $\mathcal{H}_0$ (i.e., the null hypothesis in which ξ takes on a fixed value, for instance $\xi = 0$):

$$\frac{p(\mathcal{H}_1 \mid \text{data})}{p(\mathcal{H}_0 \mid \text{data})} = \frac{p(\mathcal{H}_1)}{p(\mathcal{H}_0)} \times \frac{p(\text{data} \mid \mathcal{H}_1)}{p(\text{data} \mid \mathcal{H}_0)}. \tag{8}$$

This way of writing Bayes' rule highlights that extraordinary claims require extraordinary evidence – if a particular hypothesis $\mathcal{H}_1$ is extremely unlikely a priori, the prior odds $\frac{p(\mathcal{H}_1)}{p(\mathcal{H}_0)}$ are stacked against it, and for the posterior odds to favor $\mathcal{H}_1$ the support from the data (i.e, the degree to which $\mathcal{H}_1$ predicts $\mathcal{H}_0$) needs to be overwhelmingly strong.

Bayes' rule can also be shown to embody the *principle of parsimony*: the rule implicitly contains a preference for the simplest model that explains the data well. To see this, consider a coin with two sides and let parameter ξ indicate the chance that the coin lands heads on any throw. The null hypothesis holds that the coin is fair, with heads and tails equally likely: $\mathcal{H}_0 : \xi = 1/2$. The alternative hypothesis that we entertain here specifies that the coin may be any of the following options with equivalent likelihood: double-tails, fair, or double-heads, $\mathcal{H}_1 : \xi \in \{0, 1/2, 1\}$.

The coin is tossed and heads is observed. The probability of this datum is $1/2$ under $\mathcal{H}_0$; under $\mathcal{H}_1$, it is $p(\text{heads} \,|\, \xi = 0) \cdot p(\xi = 0 \,|\, \mathcal{H}_1) + p(\text{heads} \,|\, \xi = 1/2) \cdot p(\xi = 1/2 \,|\, \mathcal{H}_1) + p(\text{heads} \,|\, \xi = 1) \cdot p(\xi = 1 \,|\, \mathcal{H}_1) = 0 \cdot 1/3 + 1/2 \cdot 1/3 + 1 \cdot 1/3 = 1/2$. So, the datum is equally likely under $\mathcal{H}_0$ and $\mathcal{H}_1$: both models receive an equal amount of support. This

means the first outcome does not change our conviction concerning $\mathcal{H}_0$ versus $\mathcal{H}_1$. The datum did, however, change our beliefs about ξ under $\mathcal{H}_1$. Specifically, we now know that ξ cannot be zero; moreover, the datum was twice as likely under $\xi = 1$ as under $\xi = 1/2$, so that our posterior distribution for ξ under $\mathcal{H}_1$ is now $p(\xi = 1/2) = 1/3, p(\xi = 1) = 2/3$.

The coin is tossed a second time and it lands tails. Under $\mathcal{H}_0$, the probability of this happening is again $1/2$, so the total probability for the data sequence $\{\text{heads}, \text{tails}\}$ under $\mathcal{H}_0$ equals $1/2 \cdot 1/2 = 1/4$. Under $\mathcal{H}_1$, the probability of the second toss landing heads is computed under the posterior distribution obtained after the first toss, and this yields: $p(\text{tails} \,|\, \xi = 1/2) \cdot p(\xi = 1/2 \,|\, \mathcal{H}_1) + p(\text{tails} \,|\, \xi = 1) \cdot p(\xi = 1 \,|\, \mathcal{H}_1) = 1/2 \cdot 1/3 + 0 \cdot 2/3 = 1/6$. The total probability for the data sequence $\{\text{heads}, \text{tails}\}$ under $\mathcal{H}_1$ equals $1/2 \cdot 1/6 = 1/12$. This means that the observed data provided $(1/4)/(1/12) = 3$ times more support for $\mathcal{H}_0$ than for $\mathcal{H}_1$. This happens because $\mathcal{H}_1$ "spreads out" its predictions, hedging its bets. In contrast, the simple model $\mathcal{H}_0$ made a precise prediction.

Instead of learning about the predictive performance one observation at a time of $\mathcal{H}_0$ and $\mathcal{H}_1$, we could also have considered the probability that the models assign to the entire sequence $\{\text{heads}, \text{tails}\}$. Under $\mathcal{H}_0$ we again have $1/2 \cdot 1/2 = 1/4$. Under $\mathcal{H}_1$, we notice that the data falsify both $\xi = 0$ and $\xi = 1$. This leaves $\xi = 1/2$, which suggests the same answer as under $\mathcal{H}_0$, that is $1/2 \cdot 1/2 = 1/4$. However, we need to multiply this probability with $1/3$, the prior probability that $\xi = 1/2$. This is the penalty for complexity that $\mathcal{H}_1$ pays for entertaining three different values of ξ from the outset. Thus, in addition to reflecting the principle of parsimony and highlighting the utility of parsimonious explanations, Bayes' rule includes a automatic "Ockham's razor" (Jefferys & Berger, 1992; Jeffreys, 1939) in the sense that daring predictions are rewarded when they come true.

Lastly, we can also write Bayes' rule as follows:

$$\begin{aligned} p(\mathcal{H}_1 \mid \text{data}) &= \frac{p(\text{data} \mid \mathcal{H}_1) p(\mathcal{H}_1)}{p(\text{data})} \\ &= \frac{p(\text{data} \mid \mathcal{H}_1) p(\mathcal{H}_1)}{p(\text{data} \mid \mathcal{H}_1) p(\mathcal{H}_1) + p(\text{data} \mid \mathcal{H}_0) p(\mathcal{H}_0)}. \end{aligned} \tag{9}$$

This equation shows that the posterior plausibility is dictated by the predictive

performance for $\mathcal{H}_1$ and $\mathcal{H}_0$, weighted by the prior plausibility of each hypothesis. In other words, "The Bayesian world is a comparative world in which there are no absolutes." (Lindley, 2000, p. 308). This is in stark contrast to p-value statistical hypothesis testing, in which a statistical model (the null hypothesis) is judged in isolation.
